# Artificial intelligence for microembolic signal detection by transcranial Doppler in ischemic stroke: a mini-review

**DOI:** 10.3389/fneur.2026.1758938

**Published:** 2026-01-26

**Authors:** Davide Sassos, Massimo Del Sette

**Affiliations:** IRCCS Ospedale Policlinico San Martino, Genoa, Italy

**Keywords:** artificial intelligence (AI), embolic stroke of undetermined source (ESUS), ischemic stroke (IS), microembolic signals (MES), transcranial Doppler (TCD), machine learning, automated emboli detection, stroke risk stratification

## Abstract

Microembolic signals (MES) detected by transcranial Doppler (TCD) provide real-time information on ongoing embolic activity in patients with ischemic stroke and transient ischemic attack. MES have been associated with stroke recurrence and high-risk conditions including large-artery atherosclerosis, atrial fibrillation, moyamoya disease, cancer-related stroke, and complex aortic arch plaques. Despite its clinical value, conventional TCD is limited by operator dependency, suboptimal acoustic windows, and limited ability to discriminate embolus characteristics. Recent advances in artificial intelligence (AI), including machine learning algorithms and robotic-assisted TCD systems, offer automated and reproducible MES detection, improved artifact rejection, and advanced signal interpretation. This mini-review summarizes the clinical relevance of MES, the main limitations of conventional TCD, and current and emerging applications of AI to MES detection, highlighting future perspectives for stroke risk stratification and personalized secondary prevention.

## Introduction

Ischemic stroke results from cerebral arterial occlusion due to heterogeneous mechanisms, including large-artery atherosclerosis, cardioembolism, small-vessel disease, and less common vascular or hematologic disorders (1). The TOAST classification remains the most widely adopted etiological framework; however, approximately 30% of ischemic strokes are classified as cryptogenic, many of which display embolic features and are encompassed by the construct of embolic stroke of undetermined source (ESUS) (2, 3, 8).

Transcranial Doppler (TCD) enables non-invasive, bedside detection of microembolic signals (MES) within intracranial arteries, most commonly the middle cerebral artery. Unlike static imaging techniques, MES detection provides dynamic, real-time insight into embolic phenomena, offering complementary diagnostic and prognostic information in embolic stroke mechanisms.

## Microembolic signals and clinical relevance

According to established consensus criteria, MES are characterized by short duration (<300 ms), high intensity relative to background flow, unidirectionality, and a distinctive audible component (4). MES may represent solid or gaseous emboli; however, conventional TCD cannot reliably discriminate between embolus types.

**Figure 1:**
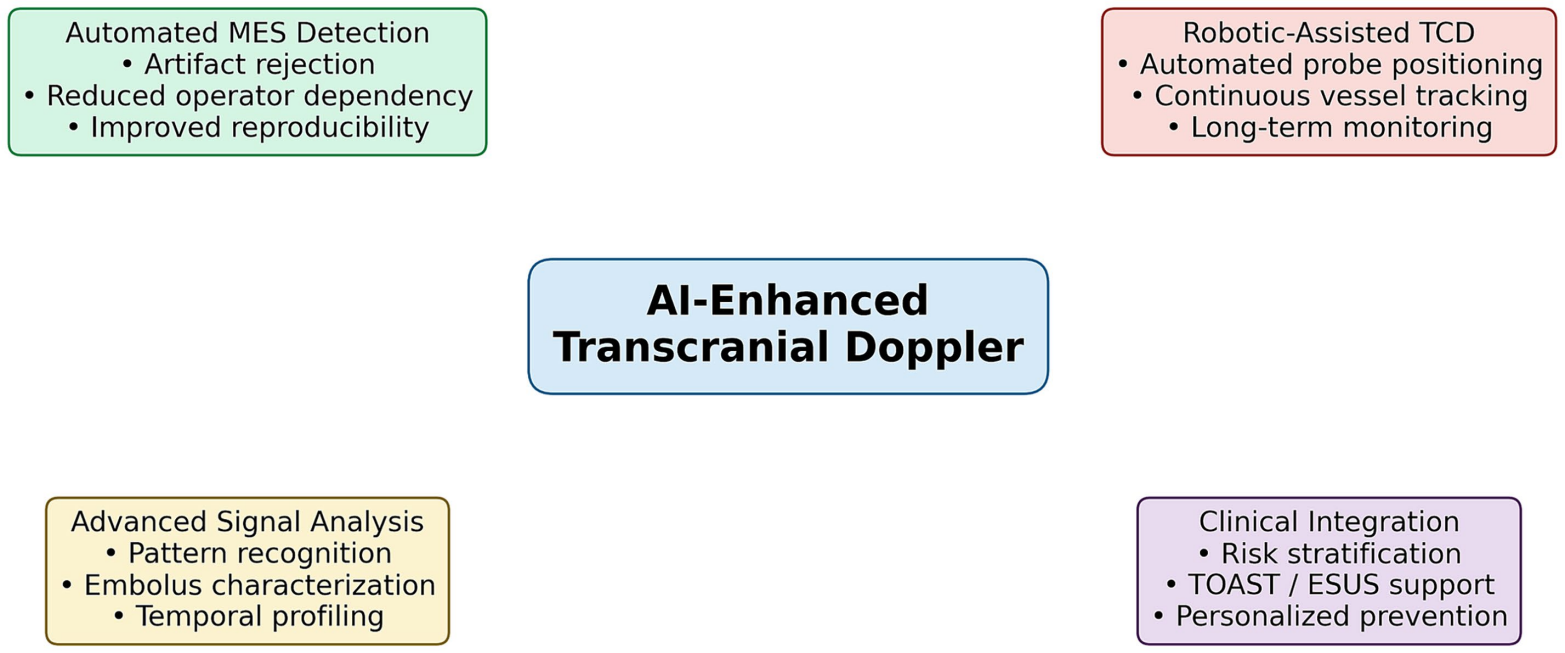
Artificial intelligence–enhanced transcranial Doppler for microembolic signal detection. Conceptual framework illustrating the main strengths of AI applications in TCD-based MES analysis. AI-driven algorithms enable automated detection of MES and discrimination from artifacts through advanced signal processing and pattern recognition. Robotic-assisted TCD systems allow automated probe positioning and continuous vessel tracking, improving signal stability and reproducibility. Advanced data analysis may support probabilistic characterization of embolus features and integration with clinical and imaging data for embolic risk stratification, etiological inference, and personalized secondary prevention strategies.

MES are more frequently detected in large-artery atherosclerosis and cardioembolic stroke, while they are uncommon in small-vessel disease. Meta-analytic data indicate a strong association between MES presence and recurrent ischemic events, supporting their role as a biomarker of ongoing embolic risk (4, 5). In selected populations—such as patients undergoing endovascular thrombectomy, those with moyamoya disease, active cancer, or complex aortic arch plaques—MES detection has been associated with worse functional outcomes and higher recurrence rates. Importantly, modulation of antithrombotic therapy has been shown to reduce or abolish MES in some settings, suggesting a potential role for MES monitoring in guiding secondary prevention strategies (Figure 1).

## Limitations of conventional TCD

Despite its advantages, conventional TCD remains highly operator-dependent and sensitive to insonation angle, patient movement, background noise, and physiological variability. Accurate MES identification requires substantial expertise, and inter-operator variability limits reproducibility across centers. In addition, conventional TCD is unable to reliably distinguish gaseous from solid emboli or to characterize the composition of solid embolic material, as Doppler signal features often overlap.

Another unresolved issue concerns the optimal duration of MES monitoring. Current protocols vary widely, ranging from short recordings to prolonged monitoring sessions, with no standardized minimum duration ensuring adequate sensitivity and specificity. These limitations have restricted the broader clinical implementation of MES monitoring despite its demonstrated prognostic value.

## Artificial intelligence applications in MES detection

Artificial intelligence offers several potential solutions to the limitations of conventional TCD. Machine learning and deep learning algorithms can be trained to automatically detect MES, improving discrimination between true embolic signals and artifacts through pattern recognition and signal feature extraction. Such automated systems may substantially reduce observer dependency and enhance diagnostic reproducibility.

Robotic-assisted TCD systems integrated with AI algorithms enable automated probe positioning and continuous vessel tracking, improving signal stability and facilitating prolonged monitoring even in uncooperative or moving patients (6). These systems may increase the feasibility of MES detection in routine clinical practice.

Advanced AI-based analysis may also allow probabilistic differentiation of embolus characteristics based on signal morphology and temporal patterns (7). In the future, MES profiles combined with clinical and imaging data could contribute to inferring stroke mechanisms, potentially supporting etiological classification frameworks such as TOAST or refining the identification of ESUS subtypes.

## Future perspectives

AI-driven TCD systems hold promise for defining standardized MES monitoring protocols, including optimization of recording duration by identifying the minimal time required to achieve reliable diagnostic performance. Integration of MES data with multimodal clinical information may further enhance individualized stroke risk stratification and guide tailored secondary prevention strategies.

Future research should focus on prospective validation of AI-based MES detection systems, assessment of their impact on clinical decision-making, and integration into stroke unit workflows. Well-designed clinical studies are required to determine whether AI-assisted MES monitoring can translate into improved outcomes for patients with embolic stroke.

## Conclusion

MES detection by TCD provides unique real-time insight into embolic activity in ischemic stroke but is limited by operator dependency, lack of standardization, and restricted embolus characterization. Artificial intelligence and robotic-assisted technologies have the potential to overcome these barriers by enabling automated, reproducible, and clinically scalable MES detection. Continued technological development and clinical validation may establish AI-enhanced TCD as a key tool in embolic stroke diagnostics and personalized secondary prevention.
